# Quantifying the Associations Between Social Determinants of Health and Blood Pressure 1–3 Years Following Pregnancy in Black Women

**DOI:** 10.1007/s40615-024-02062-5

**Published:** 2025-02-17

**Authors:** Shannon L. Walker, Rebekah J. Walker, Aprill Z. Dawson, Joni S. Williams, Anna Palatnik, Leonard E. Egede

**Affiliations:** 1 School of Medicine, Mary Ann Swetland Center for Environmental Health, Case Western Reserve University, Cleveland, OH, USA; 2 Center for Advancing Population Sciences (CAPS), Medical College of Wisconsin, Milwaukee, WI, USA; 3 Department of Medicine, Division of General Internal Medicine, The Medical College of Wisconsin, Milwaukee, WI, USA; 4 Division of Maternal-Fetal Medicine, Department of Obstetrics and Gynecology, The Medical College of Wisconsin, Milwaukee, WI, USA

**Keywords:** Social determinants of health, Blood pressure, Women’s health

## Abstract

**Objective:**

Examine the association between social determinants of health (SDOH) (cultural, neighborhood, and physical factors) and blood pressure (BP) in Black women 1–3 years following delivery.

**Methods:**

Cross-sectional data collected from *N*=204 adult Black women who had a live delivery between 2013 and 2022 in Milwaukee, Wisconsin, USA, were analyzed. Sequential linear and logistic regression models were run to test associations between SDOH variables and systolic and diastolic BP. SDOH were the independent variables; systolic and diastolic BP were the outcome variables. Stepwise linear and logistic regression with forward selection were run to assess the independent associations between SDOH and BP in Black women 1–3 after pregnancy.

**Results:**

Mean systolic and diastolic BP were 120.1 (± 17.4 SE) and 82.0 (± 13.2 SE), respectively. In final stepwise regression models, age (*β* 0.61, 95% CI 0.20, 1.02), income (*β* 5.45 95% CI 0.35, 10.56), chance health locus of control (*β* −0.78, 95% CI −1.37, −0.19), depression (*β* 0.59, 95% CI 0.10, 1.08), BMI (*β* 0.33, 95% CI 0.08, 0.57), family history of preeclampsia (*β* 7.92, 95% CI 1.49, 14.35), and kidney disease (*β* 60.32, 95% CI 38.71, 81.93) were associated with systolic BP. Age (*β* 0.53, 95% CI 0.23, 0.83), depression (*β* 0.55, 95% CI 0.22, 0.87), BMI (*β* 0.45, 95% CI 0.28, 0.63), family history of preeclampsia (*β* 5.71, 95% CI 0.97, 10.45), and kidney disease were significantly associated with diastolic BP.

**Conclusion:**

Depression was associated with increased systolic BP and chance health locus of control was associated with decreased systolic BP. Depression was significantly associated with increased diastolic BP. Future research should explore causal mechanisms between depression and hypertension risk.

Hypertension, a condition in which pressure within the blood vessels is chronically elevated (130/80 mmHg or higher), is widely prevalent among adults in the United States (US) [1–3]. As of 2021, nearly half of all US adults have diagnosed hypertension, with only 1 in 4 having controlled hypertension [1]. While hypertension has no apparent symptoms, it is an established risk factor for cardiovascular disease and stroke, which are among the leading cause of early mortality in the US [1, 4]. A large body of literature exists that has explored differences in hypertension prevalence and related complications by race and sex [5–7]. Black adults experience higher rates of hypertension compared to White adults (45.3% vs 31.4%), are more likely to develop hypertension earlier in life, and experience nearly five times greater hypertension-related mortality compared to White adults [8]. Black women have increased hypertension prevalence compared to Black men (56.9% vs 43.1%) as well as women of other racial groups, and are twice as likely to succumb to hypertension-related early mortality compared to White and Hispanic women [9, 10]. Compared to women of other racial and ethnic groups, Black women experience higher rates of blood pressure disorders during pregnancy. Specifically, the prevalence of preeclampsia among Black women is 60% higher compared to White women [6, 11]. In previously normotensive patients, preeclampsia subsequently increases risk for chronic postpartum hypertension, cardiovascular disease, and stroke [6, 11]. This risk is compounded among women with chronic hypertension who later develop preeclampsia during pregnancy [5, 12].

A multitude of factors contribute to disparities in hypertension. Often cited risk factors for increased hypertension among Black patients after delivery include increased body mass index, inadequate nutrition, and low levels of physical activity [13, 14]. However, health behavior modifications to improve blood pressure control have low uptake or are difficult to maintain long-term [15]. Recent work has identified the social determinants of health as relevant drivers of hypertension outcomes for consideration [16–20].

Social determinants of health are the conditions of the environment under which people are born, live, learn, work, play, and age that affect health, functioning, and health equity [21, 22]. A 2010 framework developed by the World Health Organization (WHO) conceptualizes social determinants of health as the interplay between two reinforcing constructs: structural and intermediary determinants of health. Included in structural determinants are larger systems-level factors that include socioeconomic and political context and socioeconomic position [21]. Social cohesion and social capital (i.e., cultural factors) act as forces that drive intermediary determinants. Within intermediary determinants are material circumstances including neighborhood factors, behaviors and biological factors, and psychosocial factors. Under this framework, populations are stratified based on their socioeconomic and political context that subsequently drive the intermediary determinants that shape one’s opportunity for optimal health and vulnerability to exposures [21]. This framework holistically considers that the immediate circumstances of individuals are driven by systems-level processes. Powell-Wiley et al. (2022) further refined this framework to incorporate relevant social processes that facilitate social exclusion and isolation of marginalized groups, including Black women and birthing groups, leading to poor outcomes in cardiovascular disease [17].

Research that has explored social determinant of health factors in relation to Black adults and hypertension risk have found inverse associations between educational attainment and hypertension risk [23]. A national cohort study identified low annual household income and educational attainment, as well as adverse neighborhood characteristics to be contributors to excess uncontrolled blood pressure among Black adults compared to White adults [19]. Qualitatively, neighborhood disorder, stress, and lack of healthy food retail have been identified as risks for hypertension [24]. Additionally, chronic psychosocial stress due to discrimination and financial strain have been identified as social risk factors for poor cardiovascular outcomes in Black women [25, 26].

Limited research has explored the association between social determinants of health and cardiovascular outcomes, including blood pressure, in Black women after pregnancy. To address this gap, the objective of this study was to understand the association between a comprehensive set of social determinants of health factors including cultural (trust, coping, resilience, and locus of control), neighborhood (perceived discrimination, crime, violence, and financial hardship), and psychosocial (depression, anxiety, and perceived stress) factors and blood pressure in a sample of adult Black women following pregnancy over and above the contribution of established clinical and demographic risk factors.

## Methods

### Study Population and Eligibility Criteria

This was a cross-sectional study of 204 adult Black participants (aged 18 years and older) from Milwaukee, Wisconsin, USA, who delivered within the Froedtert Health System between 2013 and 2022. Data collection occurred between 2019 and 2022. The Medical College of Wisconsin (MCW)’s Institutional Review Board reviewed and approved all study-related activities prior to beginning enrollment activities.

To be eligible to participate, patients needed the following: (1) be at least 18 years of age; (2) self-identify as either Black or African American; (3) be able to read and understand English; and (4) have a live birth within the Froedtert Health System between 2013 and 2022.

### Recruitment Strategies

Strategies to identify participants into this study involved use of the electronic medical record (EMR), community-based recruitment, and snowball recruitment. First, EMR-based recruitment involved compiling mailing lists from the EMR that provided contact information for all patients within the Froedtert Health System who met the age and race/ethnicity and age eligibility criteria and who delivered between 2013 and 2022. A study flyer and recruitment letter that explained the study details were mailed to the home address pulled from the EMR. Contact information for the study team was included on the study letter and flyer, and patients who were interested in participating were invited to call the number provided. If after three business days the patients had not initiated contact with the study team, they were called on the phone number provided in the medical record. The study was explained over the phone, and study appointments were scheduled for those interested in participating. Second, community-based recruitment involved placing IRB-approved study flyers in high-traffic public areas in the community such as local businesses and restaurants, grocery stores, the Milwaukee Health Department Women, Infant and Children (WIC) offices, libraries, hair salons, and clothing stores. Individuals who were interested in participating were able to call or text the study team at the number provided on the study flyer and schedule an appointment to determine if they were eligible and complete the study procedures. Lastly, snowball recruitment involved inviting participants who were enrolled in the study to share electronic and physical copies of the study flyer within their social and professional networks.

### Data Collection

First, signed informed consent was collected prior to starting any research activities. Second, while the participant was seated with both feet placed on the floor, a single blood pressure reading was taken using the Omron Blood Pressure Monitor HEM-907XL or Omron Intellisense. We followed blood pressure procedures developed by the American Heart Association for taking blood pressure readings at home [27]. When readings indicated hypertensive crisis, the medical monitor for the study was contacted for further guidance [2]. Third, measurements for height and weight were taken using a height stick and Patient Aid PA-550XL scale, respectively. Fourth, the study survey was self-administered except for instances where the participant confirmed they would like it to be read verbatim. Information on the variables for social determinants of health were obtained using a comprehensive set of survey measures previously validated in the literature.

### Variables

#### Outcome

##### Blood Pressure

Participant blood pressure was collected prior to the administration of the study survey. Participants were asked to sit quietly, with both feet flat on the floor while the blood pressure reading was obtained. The systolic and diastolic blood pressure readings were documented in the participants’ study chart immediately after the reading. The systolic reading measures the pressure in the arteries while the heart beats and the diastolic measures the pressure in the arteries while the heart rests between beats [7]. A normal blood pressure reading is a systolic at or less than 120 mmHg and a diastolic at or less than 80 mmHg [7]. Both systolic and diastolic blood pressure were treated as separate continuous measures in analyses.

#### Social Determinants of Health

The cultural factors included in this study were: trust, coping, resilience, and health locus of control. Neighborhood factors included perceived discrimination, crime, violence, and financial hardship. Psychosocial factors included depression, anxiety, and perceived stress.

#### Cultural Factors

##### Trust

Trust in health care providers was measured using a 10-item subscale from the Multidimensional Trust in Health Care System Scale (MTHCSS) where higher scores indicated greater levels of trust in health care providers [28].

##### Coping

The 35-item scale developed by Stanton et al. was used to assess two domains of coping: emotional expression (Cronbach’s alpha of 0.72) and emotional processing (Cronbach’s alpha of 0.82) [29]. Higher scores indicated greater levels of the respective coping strategy. Each subscale was treated as separate, continuous variables.

##### Resilience

The 25-item scale by Wagnild was used to measure individual resilience [30]. The scale had a Cronbach’s alpha of 0.91 and higher scores indicated greater levels of resilience. Resilience was treated as a continuous variable [30].

##### Health Locus of Control

The 18-item Multidimensional Health Locus of Control Form A scale was used to measure health locus of control [31]. The tool is comprised of three subscales: Internal Health Locus of Control, Powerful Others Health Locus of Control, and Chance Health Locus of Control. Individuals who have a higher internal health locus of control believe that their health is most strongly impacted by their own actions and ability to measure their health [31]. Those with high powerful others health locus of control believe that their health outcomes are dictated most strongly by others they deem more powerful than themselves, including but not limited to religious figures [31]. Similarly, those with high chance health locus of control believe their health is controlled by chance or fate [31]. Higher scores indicated greater locus of control for each subscale [31]. Each subscale was treated as a separate continuous variable.

#### Neighborhood Factors

##### Discrimination

A 4-item scale was used to measure experiences of discrimination based on racial or ethnic identity, sex or gender, language, or education [32]. Higher scores corresponded to higher levels of discrimination and discrimination was treated as one continuous variable.

##### Crime and Violence

The 3- and 4-item subscales of a 27-item measure developed by Echeverria were used to assess perceived exposure to neighborhood crime and violence [33]. The crime subscale had a Cronbach’s alpha of 0.77 and the violence subscale had a Cronbach’s alpha of 0.85. Each subscale was treated as separate continuous variables and higher scores corresponded to higher levels of perceived exposure to neighborhood crime and violence.

##### Financial Hardship

The 7-item Social Context Module of the Behavioral Risk Factor Surveillance System (BRFSS) was used to measure financial hardship [34]. We then created a dichotomous variable (yes or no) to indicate financial hardship.

#### Psychosocial Factors

##### Depression

The 9-item Patient Health Questionnaire (PHQ-9) was used to measure depressive symptomology [35]. The PHQ-9 had a Cronbach’s alpha of 0.89 and higher scores indicated greater levels of depressive symptomology. Depression was treated as a continuous variable [35].

##### Anxiety

The 7-item Generalized Anxiety Disorder (GAD-7) was used to assess frequency of anxiety [36]. The GAD-7 had a Cronbach’s alpha is 0.92 and higher scores corresponded to greater anxiety [36]. Anxiety was treated as a continuous variable.

##### Perceived Stress

The 4-item Perceived Stress Scale (PSS-4) was used to measure stress [37]. The PSS-4 had a Cronbach’s alpha of 0.72 and higher scores corresponded to greater levels of perceived stress [37]. Stress was treated as a continuous variable.

#### Demographic and Clinical Factors

##### Demographic Factors

Demographic factors were self-reported and included age (continuous), education (continuous), income (< $25,000 vs ≥ $25,000 annually), employment status (employed currently vs not employed currently), and marital status (dichotomized as never married and married, separated, divorced, or widowed).

##### Clinical Factors

Clinical factors were self-reported or collected from the electronic medical record. Information on body mass index (BMI), first degree relative with preeclampsia (yes vs no), preeclampsia in a previous pregnancy (yes vs no), nulliparity (yes vs no), multifetal gestation (yes vs no), and obstructive sleep apnea (yes vs no) were collected from the electronic medical record. A previous diagnosis of pregestational or gestational diabetes (yes vs no) or kidney disease (yes vs no) was based on self-report.

### Statistical Analysis

Stata SE Version 17 (StataCorp, College Station, TX) was used to perform statistical analyses. Descriptive statistics with means and standard deviations (for continuous variables) and frequencies and percentages (for categorical variables) were used to describe the sample. The outcome variables were systolic and diastolic blood pressure.

First, using the WHO conceptual framework as a guide, we ran separate sequential linear regression models for systolic blood pressure and diastolic blood pressure as outcome variables. We tested social determinants of health variables in blocks as separate models. Model 1 included demographic factors: age, education, income, employment status, and marital status. Model 2 included cultural factors: trust, emotional processing, emotional expression, resilience, internal health locus of control, chance health locus of control, and powerful others health locus of control. Model 3 included neighborhood factors: perceived discrimination, crime, violence, and financial hardship. Model 4 included psychosocial factors: depression, anxiety, and stress; and Model 5 included clinical factors: BMI, personal and family history of preeclampsia, nulliparity, multifetal gestation, and comorbid conditions (pregestational or gestational diabetes, lupus, kidney disease, and sleep apnea).

Next, stepwise linear regression models with forward selection were run to assess associations between social determinants of health, demographic, and clinical factors and blood pressure. This method was selected because it iteratively tests the large amount of predictor variables in our study regressed against a singular outcome to generate the most parsimonious final model [38, 39]. Stepwise regression with forward selection starts with an empty cell, with variables added into the model based on a predetermined *p*-value of ≤ 0.2 [39]. Stepwise regression with forward selection will only retain variables based on statistical significance, while other variables are dropped from the final models. Multicollinearity was tested using variance inflation factors (VIF). Mean VIF for systolic blood pressure was 1.28 and for diastolic blood pressure was 1.12 indicating no evidence of multicollinearity. A sensitivity analysis was run removing individuals with kidney disease to test if inclusion skewed results. There were no major differences in the magnitude of effect or size of confidence interval for either model, so results including all participants is reported. Statistical significance in the final stepwise models was determined based on a *p*-value of ≤ 0.05.

## Results

Table 1 displays the characteristics of the study sample. Overall, study participants were approximately 31 years of age (mean age of 30.9 years), and the majority were employed (73.5%), had a household income of at least $25,000 per year (62.7%), and unmarried (63.7%). The mean systolic and diastolic blood pressures for the study sample were 120.1 and 82.0, respectively.

Table 2 displays the results of the sequential linear regression models for both systolic and diastolic blood pressure. For systolic blood pressure, every year increase in age was associated with an increase of 0.81 mmHg (95% confidence interval [CI] 0.35, 1.27). Being employed was associated with an increase of 5.50 mmHg (0.09, 10.91). Each unit increase in BMI was associated with an increase of 0.4 mmHg (95% CI 0.16, 0.68), and a diagnosis of kidney disease was associated with an increase of 54.27 (95% CI 31.20, 77.34). Conversely, chance health locus of control was associated with a decrease of −0.76 mmHg (95% CI −1.41, −0.11). We found no statistically significant relationships between neighborhood or psychosocial factors and systolic blood pressure. For diastolic blood pressure, every year increase in age was associated with an increase of 0.69 (95% CI 0.34, 1.04). Every unit increase in BMI was associated with an increase of 0.50 mmHg (95% CI 0.30, 0.69), and a diagnosis of kidney disease was associated with an increase of 40.22 mmHg (95% CI 23.16, 57.27). We found no statistically significant associations between cultural, neighborhood, or psychosocial factors and diastolic blood pressure.

Table 3 displays the results of the stepwise linear regression models that examined associations between social determinants of health, demographic, and clinical factors and systolic and diastolic blood pressure. For systolic blood pressure, each year increase in age was positively associated with a 0.61 mmHg (95% CI 0.20, 1.02) increase while higher income was associated with a 5.45 mmHg (95% CI 0.35, 10.56) increase. Every unit increase in depressive symptomology was associated with a 0.59 mmHg (95% CI 0.10, 1.08) increase, while each unit increase of BMI was associated with a 0.33 mmHg (95% CI 0.08, 0.57) increase. Family history of preeclampsia was associated with a 7.92 mmHg increase, and a diagnosis of kidney disease was associated with an increase of 60.32 mmHg. Conversely, every unit increase of chance health locus of control was associated with a decrease of 0.78 mmHg (95% CI −1.37, −0.19).

For diastolic blood pressure, every year increase in age was associated with an increase of 0.53 mmHg (95% CI 0.23, 0.83). Each unit increase in depressive symptomology was associated with an increase of 0.55 mmHg (95% CI 0.22, 0.87). Every increase in BMI was associated with an increase of 0.45 mmHg (95% CI 0.28, 0.63). Family history of preeclampsia was associated with an increase of 5.71 mmHg (95% CI 0.97, 10.45), and a diagnosis of kidney disease was associated with an increase of 41.06 mmHg (95% CI 25.18, 56.94). We found no statistically significant associations between neighborhood factors and either blood pressure outcomes or cultural factors and diastolic blood pressure.

## Discussion

In our sample of 204 Black participants who were 1–3 years post-delivery from the Greater Milwaukee Area, we found demographic, cultural, psychosocial, and clinical factors associated with both systolic and diastolic blood pressure. Specifically, age and income (demographic factors), depression (psychosocial factor), and BMI, family history of preeclampsia, and kidney disease (clinical factors) were associated with increased systolic blood pressure. Whereas age (demographic factors), depression (psychosocial factor), and BMI, family history of preeclampsia, and kidney disease (clinical factor) were associated with increased diastolic blood pressure. Chance health locus of control (cultural factor) was associated with decreased systolic blood pressure

The findings of this study complement the existing research by providing estimates of the association between social determinants of health and blood pressure in Black adults after pregnancy. We found depression to be associated with both increased systolic and diastolic blood pressure in our study sample. This is supported by previous studies including a 2012 meta-analysis by Meng et al., that identified depression as a risk factor for hypertension [40]. However, the studies included in the meta-analysis were not exclusive to Black adults with a previous delivery. Studies that did include Black women were either unable to include relevant social determinants of health variables in their models or stratify by gender or maternal race and ethnicity [41–44]. Similarly, longitudinal work by Ackerman-Banks et al. (2023) that identified prenatal depression as associated with elevated risk of developing chronic hypertension 24 months postpartum among Maine births between 2007 and 2019 [45]. All births were aggregated, and the study was unable to assess differences by maternal racial or ethnic group. Given the higher rate of hypertension among Black women in the US, it is likely that the magnitude of association between prenatal depression and chronic hypertension risk would be greater for Black women than for the full sample [10]. Furthermore, the study data was restricted to insurance claims data, which inhibited the study’s ability to assess self-reported social determinants of health factors.

We found only chance health locus of control to be associated with lower blood pressure. Limited research exists that explores the association between locus of control and blood pressure. However, contrary to the findings presented in this study, earlier work by Afsahi et al. (2020) found external locus of control to be associated with increased risk of developing hypertension in a sample of Iranian adults [46]. It should be noted, however, that Afsahi’s study did not include Black women in the which may explain the conflicting findings. Given the nuances of the health locus of control construct, additional research is needed to examine chance health locus of control as a potential protective factor in hypertension risk and blood pressure control and what other factors may explain this observed relationship. Future work should also investigate health behaviors associated with health locus of control to understand how individuals’ beliefs about what drives their health outcomes influence behaviors patterns.

Interestingly, no significant associations were observed between neighborhood factors and either blood pressure outcome. One possible reason for this null finding that should be explored with additional research is the role of current racial residential segregation as a legacy of redlining. The greater Milwaukee area has been consistently identified as one of the most segregated metropolitan areas in America. Furthermore, other work has indicated that Black women are more likely to live in deprived neighborhoods compared to women of other racial groups, even after controlling for income and education [47–51]. Because of this, it is possible that within our sample there was a lack of variability among neighborhood types to observe a difference. Future research should sample participants from a range of neighborhood types and consider measures of segregation in their analyses to capture a diversity of neighborhood environments.

It should be noted that there is a body of evidence that has identified positive associations between history of preeclampsia and future chronic hypertension risk in patients [5, 12, 52]. Nevertheless, this association was not observed among this sample of Black women 1–3 years after pregnancy. It is likely that among this sample, other variables that were significantly associated with changes in blood pressure are more relevant for assessing future chronic hypertension risk and blood pressure control. Future exploratory studies are needed to better understand the specific nuances that explore this null finding in comparison to other previous work.

Of the social determinant of health variables included in this study, depression and chance health locus of control were associated with blood pressure. It is likely that among this sample of participants, collectively, clinical and demographic factors were more important drivers of blood pressure outcomes. Future work should seek to explore the interplay between depression and chance health locus of control with demographic and clinical factors and blood pressure in this population and genetic influences of hypertension risk. The findings of this study have implications for future research and practice. First, the results presented here identified significant relationships between depression and increased systolic and diastolic blood pressure. In line with research by Ackerman-Banks, additional longitudinal research that combines both self-report and clinical data is needed to explore the role of prenatal depression, including time of onset, and postpartum chronic hypertension in Black women [45]. Our finding of chance health locus of control and reduced blood pressure supports the need to also include health locus of control as a variable in this future research. Future research should also consider propensity scores as a methodological approach to assess the probability of hypertension given exposure to adverse social factors.

Second, healthcare practitioners should provide screenings for both depression and hypertension when patients seek care as well as provide integrated treatment for both depression and hypertension in patients for optimal outcomes. In an interventional study of older adults with both hypertension and depression, the intervention group who took part in integrated depression and hypertension outpatient care were more likely to report improved depression scores, lower blood pressure, and greater adherence to antidepressant and antihypertensive medication [53]. Additional prospective and longitudinal studies are needed to explore other confounding factors that may affect this relationship. Furthermore, prenatal assessment and management of depression should be considered as a means of optimizing outcomes later in life during pregnancy.

The primary strength of our work is the use of prospective data collection to assess multiple social determinants of health and their associations with blood pressure in Black women. However, there are a few limitations to consider. This study used a cross-sectional design, which prohibits any assumptions to be made about causality. Participants for this study were recruited from one health system in the Greater Milwaukee Area which may limit the generalizability of the findings presented here. We assessed social determinants of health factors 1 to 3 years following delivery. It is possible that some factors have changed over time and that is not reflected within this dataset. Blood pressure readings were collected during one study visit and may not capture possible variations in blood pressure readings over time. Lastly, there is the potential for selection bias among our sample wherein we included only participants who had a live delivery and did not include those who experienced a stillbirth, miscarriage, or terminated pregnancy which may further limit generalizability.

## Conclusion

This is one of the first studies to assess associations between several social determinants of health and blood pressure in a sample of Black women with a previous delivery. In addition to known clinical and demographic risk factors, we found significant relationships between chance health locus of control and systolic blood pressure and depression and systolic and diastolic blood pressure. The results provide preliminary insight on the associations between the social determinants of health and blood pressure in Black women. Future work should focus on integrating robust clinical and self-report data and include integrated interventions for best outcomes.

## Figures and Tables

**Table 1:** Sample demographics for the study sample of adult black postpartum women

Variables	Percent or mean ± standard deviation (SD) *n*=204

Age (years)	30.9 ± 5.6
Education (years)	14.6 ± 2.7
Income	
<$25,000	37.3%
≥$25,000	62.7%
Employment status	
Not employed	26.5%
Employed	73.5%
Marital status	
Never married	63.7%
Married/separated/divorced/widowed	36.3%
Cultural factors	
Provider trust	36.4 ± 8.1
Emotional processing	12.0 ± 2.7
Emotional expression	11.8 ± 3.3
Resilience	145.3 ± 19.6
Locus of control, internal	20.8 ± 4.5
Locus of control, chance	13.3 ± 4.0
Locus of control, others	14.3 ± 3.9
Neighborhood factors	
Perceived discrimination	3.0 ± 1.7
Crime	2.5 ± 0.84
Violence	6.3 ± 3.0
Financial hardship	
No	72.6%
Yes	27.4%
Psychosocial factors	
Depression	6.5 ± 5.5
Anxiety	6.5 ± 5.5
Perceived stress	6.3 ± 3.4
Clinical factors	
BMI (kg/m^2^)	32.9 ± 8.9
Family history of preeclampsia	
No	86.3%
Yes	13.7%
History of preeclampsia	
No	85.3%
Yes	14.7%
Nulliparity (yes)	
No	67.5%
Yes	32.5%
Multifetal gestation (yes)	
No	95.1%
Yes	4.9%
Diabetes	
No	89.2%
Yes	10.8%
Lupus	
No	99.0%
Yes	1.0%
Kidney disease	
No	99.0%
Yes	1.0%
Obstructive sleep apnea	
No	98.5%
Yes	1.5%
Clinical outcomes	
Mean systolic blood pressure	120.1 ± 17.4
Mean diastolic blood pressure	82.0 ± 13.2

**Table 2:** Sequential unadjusted linear models for the association between blood pressure and the social determinants of health (demographics, cultural, neighborhood, psychosocial, and clinical factors)

	Systolic blood pressure Coefficient (95% CI)	Diastolic blood pressure Coefficient (95% CI)

Model 1: demographics		
Age	0.81 (0.35, 1.27)**	0.69 (0.34, 1.04)***
Education	−0.54 (−1.53, 0.44)	−0.35 (−1.10, 0.40)
Income		
≥$25,000 (ref: <$25,000)	3.20 (−2.37, 8.78)	2.10 (−2.13, 6.33)
Employment		
Employed (ref: unemployed)	5.50 (0.09, 10.91)	1.91 (−2.19, 6.02)
Marital status		
Married/separated/divorced/widowed (ref: never married)	−1.08 (−6.56, 4.41)	−0.61 (−4.77, 3.55)
Model 2: cultural factors		
Provider trust	0.13 (−0.19, 0.45)	0.12 (−0.13, 0.37)
Emotional processing	−0.37 (−1.75, 1.01)	0.35 (−0.71, 1.40)
Emotional expression	−0.03 (−1.10, 1.04)	−0.25 (−1.07, 0.57)
Resilience	0.08 (−0.08, 0.24)	0.01 (−0.10, 0.13)
Locus of control, internal	0.12 (−0.43, 0.68)	−0.1 (−0.53, 0.33)
Locus of control, chance	−0.76 (−1.41, −0.11)*	−0.44 (−0.93, 0.06)
Locus of control, others	0.55 (−0.15, 1.26)	0.21 (−0.33, 0.75)
Model 3: neighborhood factors		
Perceived discrimination	0.17 (−1.35, 1.70)	0.44 (−0.72, 1.60)
Crime	1.45 (−2.37, 5.27)	−0.34 (−3.24, 2.56)
Violence	−0.16 (−1.23, 0.91)	0.07 (−0.75, 0.89)
Financial hardship (ref: no financial hardship)	1.19 (−4.53, 6.92)	0.81 (−3.55, 5.16)
Model 4: psychosocial factors		
Depression	0.58 (−0.15, 1.32)	0.47 (−0.09, 1.02)
Anxiety	−0.42 (−1.19, 0.34)	−0.21 (−0.79, 0.36)
Stress	−0.20 (−1.10, 0.70)	−0.32 (−1.00, 0.36)
Model 5: clinical factors		
Body mass index	0.42 (0.16, 0.68)**	0.50 (0.30, 0.69)***
Family history of preeclampsia (yes)	4.99 (−1.65, 11.64)	2.82 (−2.09, 7.73)
History of preeclampsia (yes)	3.35 (−3.46, 10.16)	2.45 (−2.56, 7.51)
Nulliparity (yes)	−2.20 (−7.30, 2.89)	−0.29 (−4.05, 3.48)
Multifetal gestation (yes)	−0.79 (−11.43, 9.85)	0.56 (−7.31, 8.43)
Diabetes	2.19 (−5.35, 9.73)	3.40 (−2.17, 8.98)
Lupus	−1.31 (−24.17, 21.56)	−0.44 (−17.34, 16.46)
Kidney disease	54.27 (31.20, 77.34)***	40.22 (23.16, 57.27)***
Obstructive sleep apnea	11.57 (−7.59, 30.74)	2.14 (−12.02, 16.30)

Significance at

*** *p*<0.001

** *p*<0.01

* *p*<0.05

**Table 3:** Stepwise linear models for the association between blood pressure and the social determinants of health (demographics, cultural, neighborhood, and clinical factors)

	Systolic blood pressure Coefficient (95% CI)	Diastolic blood pressure Coefficient (95% CI)

Demographics		
Age	0.61 (0.20, 1.02)**	0.53 (0.23, 0.83)**
Education	−0.82 (−1.75, 0.11)	--
Income	--	--
≥$25,000	5.45 (0.35, 10.56)*	2.33 (−1.14, 5.81)
Employment	--	--
Employed	3.83 (−1.22, 8.88)	--
Marital status	--	--
Married/separated/divorced/widowed	--	--
Cultural factors	--	--
Trust	0.21 (−0.1, 0.50)	0.19 (−0.01, 0.39)
Emotional processing	--	0.52 (−0.11, 1.16)
Emotional expression	--	--
Resilience	0.09 (−0.05, 0.23)	--
Locus of control internal	--	--
Locus of control chance	−0.78 (−1.37, −0.19)**	−0.33 (−0.74, 0.09)
Locus of control others	0.51 (−0.10, 1.13)	--
Neighborhood factors	--	--
Perceived discrimination	--	--
Crime	--	--
Violence	--	--
Financial hardship	3.29 (−1.70, 8.30)	--
Psychosocial factors	--	--
Depression	0.59 (0.10, 1.08)*	0.55 (0.22, 0.87)**
Anxiety	--	--
Stress	--	--
Clinical factors	--	--
Body mass index	0.33 (0.08, 0.57)**	0.45 (0.28, 0.63)***
Family history of preeclampsia	7.92 (1.49, 14.35)*	5.71 (0.97, 10.45)*
History of preeclampsia	--	--
Nulliparity	--	--
Multifetal gestation	--	--
Diabetes	----	--
Lupus	--	--
Kidney disease	60.32 (38.71, 81.93)***	41.06 (25.18, 56.94)***
Obstructive sleep apnea	--	--

Significance at

*** *p*<0.001

** *p*<0.01

* *p*<0.05

Text boxes with -- indicate variables that were dropped from the stepwise regression models based on level of statistical significance

## Data Availability

Data may be accessible by contacting the corresponding author.
